# Steroid-Induced Central Serous Chorioretinopathy in Uveitis: Diagnostic Challenges and Multimodal Imaging with a Primary Inflammatory Choriocapillaropathy Illustrative Case

**DOI:** 10.3390/diagnostics16162679

**Published:** 2026-08-21

**Authors:** Maria Carmela Saturno, Claudia Smarra, Maurizio La Cava, Oscar Matteo Gagliardi, Alessandro Ansevini, Rosalia Giustolisi, Alessandro Lambiase, Danilo Iannetta

**Affiliations:** Department of Sense Organs, Sapienza University of Rome, 00161 Rome, Italy; mariacarmela.saturno@uniroma1.it (M.C.S.); claudia.smarra@uniroma1.it (C.S.); maurizio.lacava@uniroma1.it (M.L.C.); oscarmatteo.gagliardi@uniroma1.it (O.M.G.); ansevini.2281268@studenti.uniroma1.it (A.A.); rosalia.giustolisi@uniroma1.it (R.G.); alessandro.lambiase@uniroma1.it (A.L.)

**Keywords:** central serous chorioretinopathy, uveitis, corticosteroids, ampiginous choroiditis, relentless placoid chorioretinitis, multimodal imaging, choriocapillaris

## Abstract

**Background and Clinical Significance.** Steroid-induced central serous chorioretinopathy (CSCR) is a potential complication in patients with uveitis receiving corticosteroid therapy and may be misinterpreted as persistent inflammatory activity. Inflammatory and iatrogenic subretinal fluid may coexist, particularly in primary inflammatory choriocapillaropathies (PICCPs), creating diagnostic and therapeutic challenges. This narrative review discusses the pathophysiological mechanisms underlying steroid-induced CSCR and emphasizes the value of multimodal imaging in distinguishing inflammatory subretinal fluid from steroid-induced serous retinal detachment. Optical coherence tomography, fundus autofluorescence, fluorescein angiography and indocyanine green angiography provide complementary information that may guide accurate diagnosis and avoid inappropriate corticosteroid escalation. **Case Presentation.** An illustrative case of ampiginous choroiditis, a severe form of PICCP, complicated by bilateral steroid-induced CSCR shortly after initiation of high-dose corticosteroid therapy, is presented to highlight the clinical relevance of this complication and its therapeutic implications. **Conclusions.** Early recognition through multimodal imaging enabled prompt corticosteroid tapering and introduction of steroid-sparing immunosuppression, with subsequent marked anatomical and functional improvement.

## 1. Introduction

Primary inflammatory choriocapillaropathies (PICCPs), also referred to as choriocapillaritis, are posterior uveitis characterized by primary involvement of the choriocapillaris, leading to areas of hypoperfusion and subsequent damage to the retinal pigment epithelium (RPE) and outer retina [1]. Within this spectrum, ampiginous choroiditis, also known as relentless placoid chorioretinitis, is a rare and potentially vision-threatening entity that predominantly affects young adults. It is characterized by multifocal placoid chorioretinal lesions extending from the posterior pole to the peripheral fundus and may follow a chronic course requiring systemic immunosuppression [2].

Systemic corticosteroids remain the cornerstone of first-line therapy during the acute phase of PICCPs, particularly in patients with significant visual impairment due to posterior pole involvement [1,3]. However, corticosteroid exposure is a well-recognized risk factor for the development of central serous chorioretinopathy (CSCR), a serous detachment of the neurosensory retina [4,5,6].

In patients with uveitis, particularly PICCPs, the occurrence or worsening of subretinal fluid during steroid treatment is a significant diagnostic challenge, as it may reflect either persistent inflammation or the development of iatrogenic CSCR. Misinterpretation of this complication may lead to inappropriate escalation of corticosteroid therapy and further worsening of the underlying condition [4,7].

This narrative review discusses the pathophysiological mechanisms and multimodal imaging findings of steroid-induced CSCR in uveitic patients, with particular focus on PICCPs. An illustrative case of ampiginous choroiditis complicated by bilateral steroid-induced CSCR is presented to emphasize the clinical relevance of this complication and its therapeutic implications.

## 2. Pathophysiology of Steroid-Induced Central Serous Chorioretinopathy

CSCR is a disorder within the pachychoroid disease spectrum [8]. First described by Albrecht von Graefe in 1866 [9], CSCR predominantly affects individuals of working age, with incidence rates peaking at approximately 70 cases per 100,000 person-years in the 40–49-year age group, and a significantly higher risk in males [10]. Its pathophysiology involves dysregulation of choroidal circulation, leading to impaired RPE pump function and accumulation of subretinal fluid. Increasing evidence highlights a central role of mineralocorticoid receptor activation within the choroidal vasculature, promoting vasodilation and hyperpermeability, key features of the pachychoroid phenotype [8,11].

Corticosteroid exposure represents one of the most consistently identified risk factors for CSCR. Both endogenous hypercortisolism and exogenous corticosteroid administration have been associated with disease onset, persistence, exacerbation and recurrence, regardless of the route of administration. Compared with idiopathic forms, corticosteroid-associated CSCR is characterized by a less marked male predominance and a higher prevalence of bilateral and atypical presentations, suggesting a distinct clinical phenotype [11,12].

The mechanisms underlying steroid-induced CSCR are multifactorial and not yet fully elucidated. Glucocorticoids may alter choroidal vascular autoregulation, enhance catecholamine-mediated vascular instability and exert mineralocorticoid-like effects, contributing to vascular fragility and increased permeability [11]. In parallel, corticosteroids directly impair RPE function by altering electrophysiological properties and ion transport, thereby reducing subretinal fluid reabsorption [13]. Additional mechanisms may include potentiation of epinephrine-induced vascular effects, RPE apoptosis and glucocorticoid-induced changes in blood rheology, promoting choroidal congestion and microvascular dysfunction [12,14,15].

Experimental data further support the role of mineralocorticoid receptor activation in this process, as aldosterone has been shown to induce choroidal thickening, vascular leakage and structural alterations of RPE cells, providing a biological rationale for the use of mineralocorticoid receptor antagonists in selected cases [12].

## 3. Diagnostic Pitfalls in PICCPs: Distinguishing Inflammatory Activity from Steroid-Induced Serous Retinal Detachment

Multimodal imaging is essential for differentiating inflammatory activity from steroid-induced CSCR in PICCPs. Imaging abnormalities in these disorders result from inflammatory choriocapillaris ischemia with secondary injury to the RPE and outer retina. The severity and distribution of these lesions vary across the PICCP spectrum, ranging from subtle end-capillary involvement in multiple evanescent white dot syndrome (MEWDS) to larger and deeper areas in acute posterior multifocal placoid pigment epitheliopathy (APMPPE), multifocal choroiditis (MFC), serpiginous choroiditis (SC) and ampiginous choroiditis [2,16,17].

Optical coherence tomography (OCT) is usually the first-line imaging modality in clinical practice. In active PICCPs, OCT demonstrates disruption of the ellipsoid zone and photoreceptor outer segments, hyperreflective outer retinal changes, focal RPE alterations and variable retinal thickening. In more severe entities, including APMPPE, SC and ampiginous choroiditis, subretinal fluid and intraretinal edema may also occur [16,17,18]. In ampiginous choroiditis, additional findings may include superficial choroidal hyperreflectivity, focal RPE detachments, and, in advanced stages, bacillary layer detachment, a feature that may closely resemble CSCR-related changes. Hyperreflective vitreous dots consistent with inflammatory cells may further support active disease [2]. Conversely, CSCR presents with dome-shaped serous neurosensory retinal detachment, associated with pachychoroid features on enhanced-depth imaging OCT (EDI-OCT) [19].

OCT angiography (OCTA) may provide additional information in this differential diagnosis. In active ampiginous choroiditis, choriocapillaris flow deficits colocalize with active placoid lesions and with the corresponding hypofluorescent areas on ICGA, whereas flow tends to recover as lesions become inactive [2]. In CSCR, OCTA may demonstrate heterogeneous choriocapillaris perfusion abnormalities, including areas of reduced flow signal with adjacent hyperperfusion [19]. The main additional value of OCTA is the detection of secondary choroidal neovascularization, as it can directly delineate the neovascular flow network [19].

Fundus autofluorescence (FAF) reflects metabolic alterations of the RPE. In active PICCP lesions, hyperautofluorescence is associated with lipofuscin accumulation within damaged RPE secondary to choriocapillaris ischemia, whereas hypoautofluorescence corresponds to RPE atrophy [20]. In ampiginous choroiditis, characteristic “cockade” lesions with concentric rings of alternating hypo- and hyperautofluorescence may be observed during the active stages [21]. In CSCR, acute lesions may appear hypoautofluorescent because of masking by subretinal fluid, while chronic disease commonly demonstrates granular RPE alterations [19].

Fluorescein angiography (FA) has a complementary role in PICCPs. Active lesions may show early hypofluorescence followed by late staining or early hyperfluorescence secondary to RPE atrophy [16,17,18]. In ampiginous choroiditis, FA typically demonstrates multiple confluent hypofluorescent placoid lesions in the early phases, followed by late fluorescein leakage involving both the chorioretinal lesions and, in severe inflammatory stages, the optic disc [2]. In contrast, CSCR classically demonstrates focal RPE leakage with “inkblot” or “smokestack” patterns [19].

Indocyanine green angiography (ICGA) remains the most informative imaging modality for evaluating PICCPs and is particularly useful for identifying inflammatory choriocapillaris hypoperfusion. It demonstrates patchy or geographic hypofluorescent areas, frequently extending beyond clinically visible lesions [16,17,22,23]. In ampiginous choroiditis, lesions commonly involve both the posterior pole and peripheral fundus, forming confluent geographic hypofluorescent areas consistent with deep choriocapillaris involvement [2]. By contrast, ICGA in CSCR demonstrates focal or multifocal areas of choroidal hyperpermeability related to pachychoroid vascular congestion [19].

Overall, PICCPs are characterized by inflammatory choriocapillaris hypoperfusion associated with secondary outer retinal ischemic damage, whereas CSCR predominantly reflects pachychoroid-related choroidal hyperpermeability with serous retinal detachment. A summary of the principal multimodal imaging findings is provided in Table 1.

## 4. Illustrative Case

A 48-year-old Caucasian male presented to the Ocular Immunovirology Unit at Sapienza University of Rome in September 2025 with a six-month history of progressive bilateral visual impairment. Best-corrected visual acuity (BCVA) was 20/33 in the right eye and 20/100 in the left eye. Anterior segment examination was unremarkable in both eyes.

**Figure 1:**
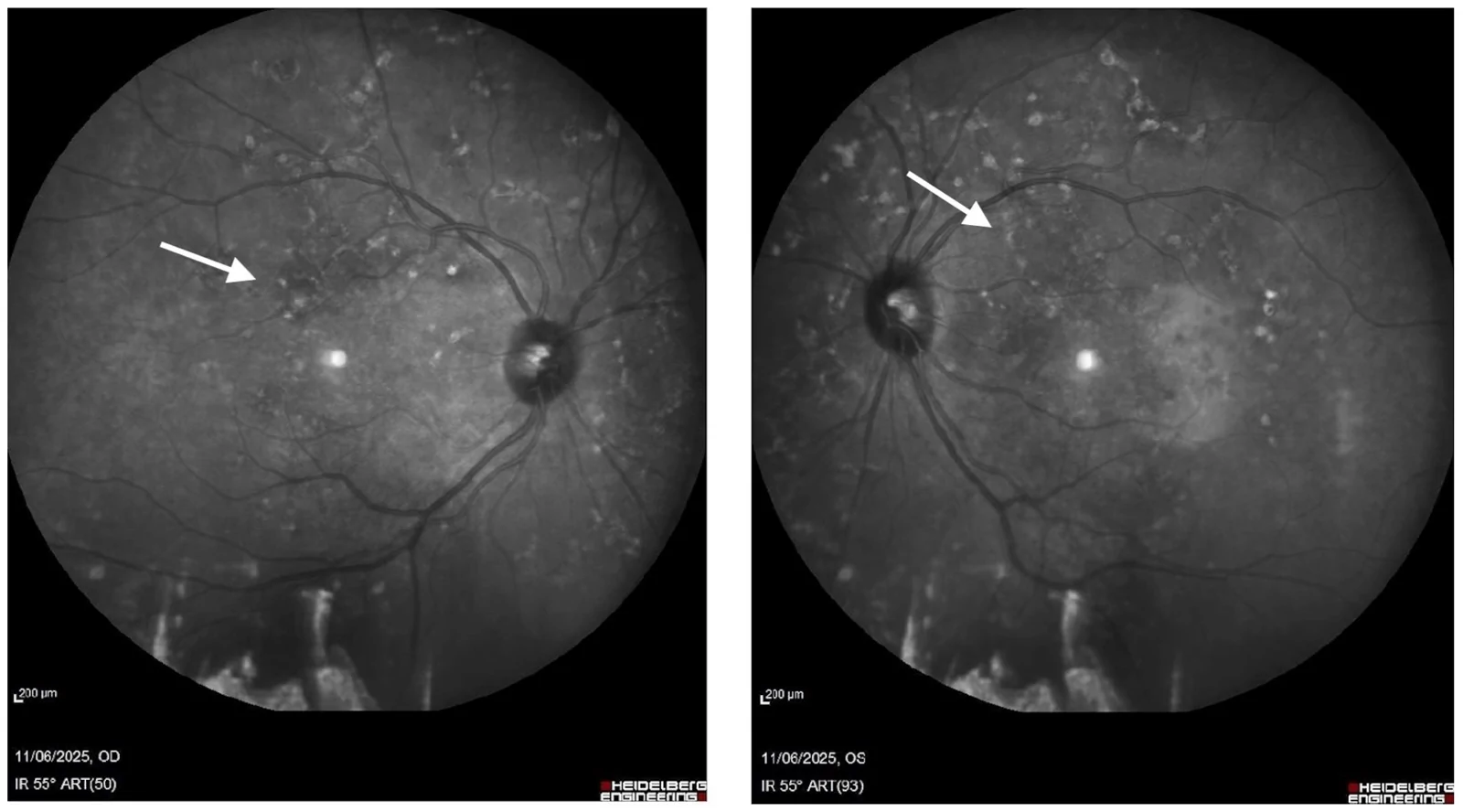
Ultra-widefield infrared fundus images of both eyes, showing hypoautofluorescent lesions surrounded by hyperautofluorescent halos (arrow).

Dilated fundus examination revealed multiple yellow-white placoid lesions involving the posterior pole and peripapillary area bilaterally. Multimodal imaging was performed to further characterize these findings. OCT showed disruption of the outer retinal layers with RPE alterations. FAF showed scattered hypoautofluorescent lesions with hyperautofluorescent borders, including characteristic “cockade” lesions (Figure 1).

**Figure 2:**
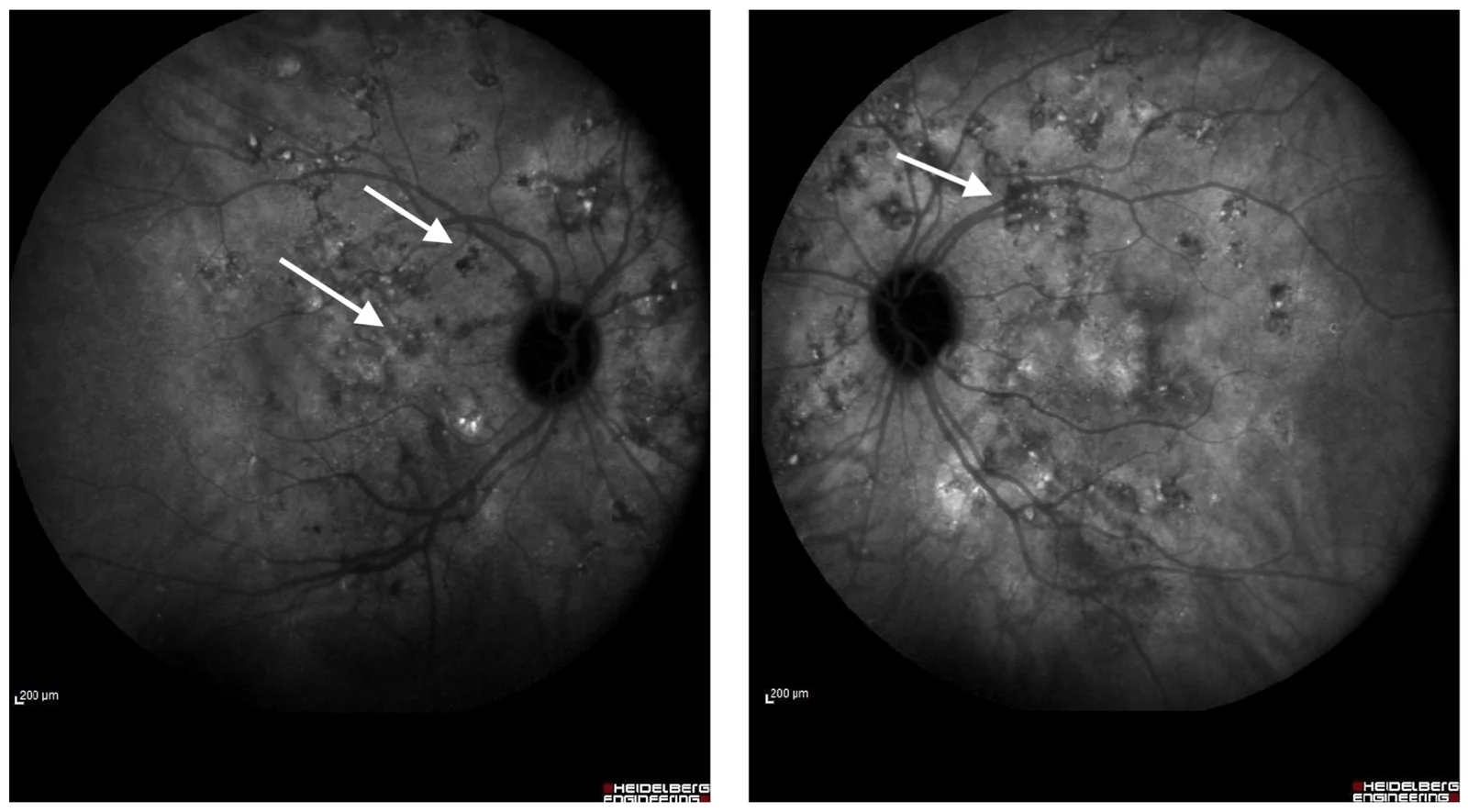
Mid-phase Indocyanine Green Angiography (ICGA) of both eyes, showing multiple, disseminated hypofluorescent spots, consistent with activechoriocapillaritis lesions (arrows).

FA demonstrated early hypofluorescent dots with late staining, while ICGA revealed persistent hypofluorescent areas throughout all phases, consistent with choriocapillaris hypoperfusion (Figure 2).

Infectious etiologies were excluded through comprehensive laboratory testing and neuroimaging findings were unremarkable. Based on the clinical and multimodal imaging findings, a diagnosis of ampiginous choroiditis was established. Systemic corticosteroid therapy with oral prednisone (1 mg/kg/day) was initiated to control inflammatory activity.

**Figure 3:**
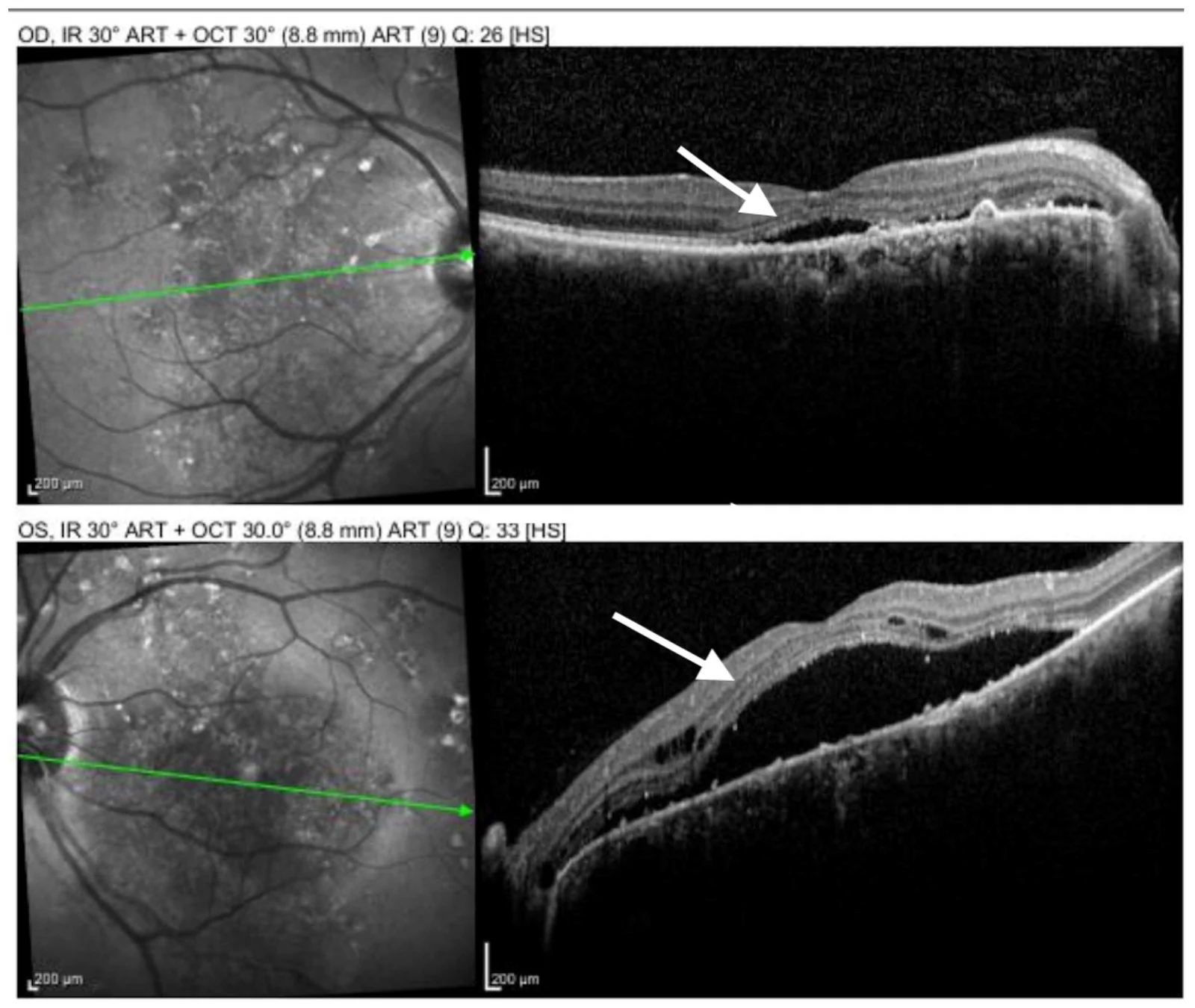
SD-OCT of both eyes 10 days after initiating steroid treatment, showing dome-shaped serous detachment of the neurosensory retina (arrows).

Ten days after treatment initiation, the patient reported acute bilateral visual worsening with chromatopsia. Fundus examination revealed serous macular detachment in both eyes, and OCT confirmed dome-shaped neurosensory retinal detachment (Figure 3). FA demonstrated focal “inkblot” leakage, while ICGA showed choroidal hyperpermeability superimposed on the pre-existing hypofluorescent areas. Recurrent ampiginous choroiditis was not supported by the absence of new creamy placoid lesions, early hypofluorescent lesions with late leakage on FA, or new persistent hypofluorescent areas of choriocapillaris hypoperfusion on ICGA. A VKH-like inflammatory serous detachment was also excluded from the leading differential, as ICGA lacked the diffuse stromal inflammatory pattern typical of VKH, while FA showed focal inkblot leakage rather than multiple pinpoint leaks with subretinal pooling. Choroidal neovascularization was similarly unsupported, as FA showed no early iso- or hyperfluorescent lesion with progressive late leakage and ICGA revealed no branching choroidal neovascular complex. Overall, the close temporal relationship with high-dose corticosteroid therapy and the multimodal imaging pattern were consistent with steroid-induced CSCR.

Systemic corticosteroids were promptly tapered, oral canrenone (100 mg/day) was initiated as adjunctive treatment, and mycophenolate mofetil (2 g/day) was introduced as steroid-sparing immunosuppressive therapy. Serum potassium levels and renal function were monitored during canrenone treatment and remained within normal limits.

At two-month follow-up, multimodal imaging demonstrated near-complete resolution of subretinal fluid, with restoration of normal foveal contour and reconstitution of the outer retinal layers. ICGA confirmed stabilization of previously hypoperfused areas without evidence of new inflammatory foci. BCVA improved to 20/25 in the right eye and 20/40 in the left eye. Oral canrenone was therefore discontinued.

**Figure 4:**
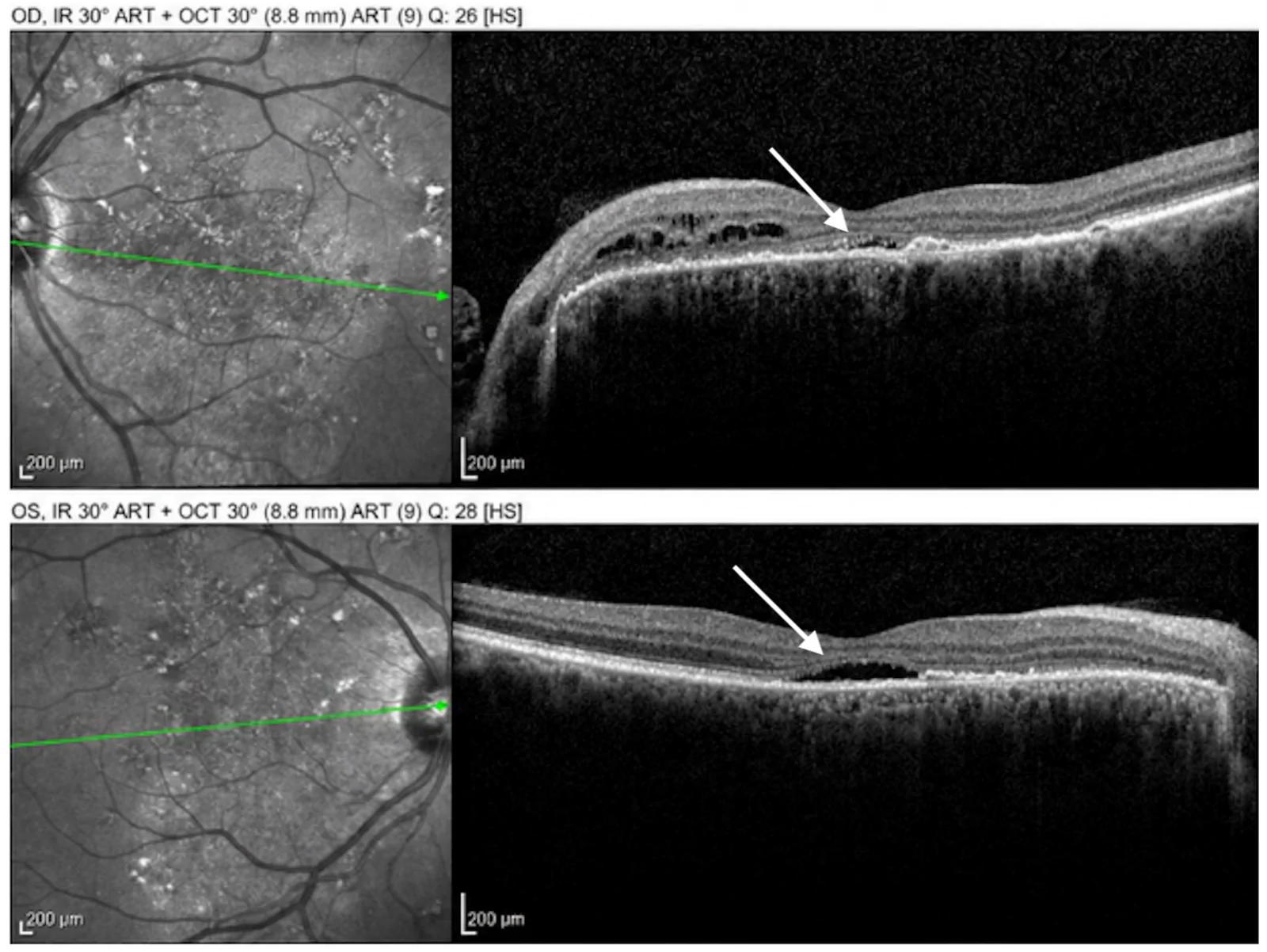
At the final follow-up, the serous neurosensory retinal detachment had significantly regressed (arrows) .

At the latest follow-up in March 2026, BCVA further improved to 20/20 in the right eye and 20/22 in the left eye, with stable multimodal imaging findings and no evidence of disease recurrence. The patient showed good tolerance to mycophenolate mofetil, without reported adverse effects (Figure 4).

## 5. Discussion

To the best of our knowledge, this is the first reported case of ampiginous choroiditis complicated by steroid-induced CSCR shortly after initiation of systemic corticosteroid therapy, highlighting the diagnostic and therapeutic challenge posed by new subretinal fluid in this setting.

The occurrence of iatrogenic CSCR has been reported across a range of uveitic disorders. Khairallah et al. described a series of 14 patients (20 eyes) who developed CSCR following corticosteroid treatment, including cases of Behçet uveitis, birdshot chorioretinopathy, ocular toxoplasmosis and HLA-B27-associated uveitis. In their cohort, which was predominantly male (78.6%), corticosteroids were administered via different routes, most commonly oral prednisone at doses ranging from 5 to 80 mg/day, as well as intravenous methylprednisolone and periocular triamcinolone. CSCR was both unilateral and bilateral, with characteristic focal leakage on FA observed in 85% of cases. Management primarily consisted of corticosteroid tapering or discontinuation, resulting in resolution of subretinal fluid in most eyes, while only one case required additional treatment with focal laser photocoagulation [12].

Dutta Majumder et al. reported a retrospective series of 26 patients (31 eyes), predominantly male (88.4%), who developed CSCR in the setting of uveitis. The mean age was 42.8 years and bilateral involvement was observed in 19.2% of cases. Most patients were receiving systemic corticosteroid therapy at the time of CSCR onset (88.5%). Choroiditis was the most frequent underlying condition (48.4%), including serpiginous and serpiginous-like (tubercular) forms and Vogt–Koyanagi–Harada disease. Other associated uveitis included retinal vasculitis, anterior uveitis, scleritis and ocular toxoplasmosis. Multimodal imaging, including FA and OCT, was used to confirm the diagnosis. Management was primarily based on corticosteroid reduction or discontinuation, often combined with immunosuppressive therapy, while focal laser photocoagulation was required in 29% of eyes and photodynamic therapy in one case. Compared with our case, CSCR showed a more variable course, with a longer time to resolution (mean 99 ± 61 days). Importantly, in some patients, initial misinterpretation of subretinal fluid as persistent inflammatory activity led to corticosteroid escalation, resulting in worsening of the underlying CSCR [4].

Schalenbourg et al. described three patients (two males and one female) with chronic inflammatory ocular diseases, including birdshot chorioretinopathy, Vogt–Koyanagi–Harada disease and diffuse scleritis associated with relapsing polychondritis. CSCR was unilateral in all cases and the diagnosis was confirmed by multimodal imaging. Unlike our case, CSCR developed after prolonged corticosteroid exposure in the setting of long-standing disease. Although corticosteroid tapering represented the main therapeutic approach, two of the three patients required additional focal laser photocoagulation because of persistent CSCR, in combination with steroid-sparing immunosuppressive therapy such as cyclosporine or cyclophosphamide [24].

Gupta et al. reported four patients (three males and one female) with ocular inflammatory diseases, including juvenile idiopathic arthritis-associated uveitis, recurrent anterior uveitis in Addison’s disease, inflammatory bowel disease-associated sclerokeratitis and sarcoidosis-associated uveitis. CSCR was unilateral in all cases, although one patient had a history of bilateral involvement. In contrast to our case, CSCR was not limited to treatment onset but showed a variable course, including recurrence and worsening with continued corticosteroid exposure. Notably, in one patient, visual deterioration following prednisone initiation led to an initial misinterpretation as active disease, with subsequent improvement only after steroid withdrawal. Management primarily relied on corticosteroid discontinuation, with spontaneous resolution in most cases, while focal laser photocoagulation was required in one patient [7].

Baumal et al. described CSCR in a 37-year-old male following a single periocular (sub-Tenon) triamcinolone injection administered for HLA-B27-associated iritis. CSCR developed within one week of local corticosteroid administration, highlighting that even this route may trigger this complication. This case reinforces the need to consider CSCR even in the absence of systemic corticosteroid therapy [25].

Takayama et al. reported chronic Vogt–Koyanagi–Harada disease complicated by corticosteroid-induced CSCR and systemic steroid-related adverse effects. The authors introduced adalimumab, allowing discontinuation of corticosteroids and cyclosporine, with subsequent control of both inflammation and CSCR [26].

Özdemir et al. reported CSCR in a patient with Behçet’s uveitis receiving systemic corticosteroids together with cyclosporine and azathioprine. In that case, corticosteroids were discontinued, and infliximab was introduced to control the underlying inflammatory disease. However, subretinal fluid persisted despite steroid withdrawal, and half-fluence photodynamic therapy was required, resulting in complete resolution of the subfoveal fluid and visual improvement. This case shows that in uveitic patients with posterior segment involvement, steroid withdrawal alone may be insufficient and CSCR-directed treatment may be necessary [27].

Compared with previous reports, our case expands the spectrum of steroid-induced CSCR in uveitis by documenting its occurrence in ampiginous choroiditis, a PICCP in which systemic corticosteroids represent the first-line therapy due to their prompt anti-inflammatory effect [2]. The rapid onset of bilateral serous retinal detachment shortly after high-dose prednisone, together with the transition from choriocapillaris hypoperfusion to choroidal hyperpermeability on multimodal imaging, supported the diagnosis of steroid-induced CSCR.

Ampiginous choroiditis is a severe and recurrent primary inflammatory choriocapillaropathy characterized by multiple placoid lesions at different stages of activity. Active lesions are associated with choriocapillaris hypoperfusion, appearing hypofluorescent on early FA with late staining, persistently hypofluorescent on ICGA, and showing corresponding outer retinal changes on OCT [2,16]. Subretinal fluid and, less frequently, intraretinal fluid may also occur during active disease, further complicating the interpretation of newly developed retinal fluid on OCT. Therefore, in patients receiving corticosteroid therapy, the appearance of new subretinal fluid may reflect either recurrent inflammatory activity or steroid-induced CSCR.

Given the frequent refractoriness of ampiginous choroiditis to corticosteroid monotherapy, early introduction of corticosteroid-sparing immunosuppressive therapy, with the addition of biologic agents, when necessary, is generally advocated to prevent the extensive, vision-threatening chorioretinal scarring associated with uncontrolled disease. However, this aggressive therapeutic strategy, together with the unavoidable reliance on systemic corticosteroids as first-line treatment, may expose patients to an increased risk of steroid-related complications, including CSCR, as observed in the present case [2,16].

In this setting, our decision to intervene promptly at the onset of CSCR was motivated by the need to preserve visual function while avoiding irreversible outer retinal damage that may result from persistent subretinal fluid accumulation or insufficient control of the underlying inflammatory activity.

In the present case, management was primarily guided by the acute and corticosteroid-induced nature of the CSCR episode. Prompt tapering of systemic corticosteroids represented the main therapeutic intervention, while mycophenolate mofetil was introduced to maintain control of the underlying inflammatory disease and enable steroid reduction. This approach differs from that generally adopted for persistent or chronic CSCR, for which reduced-dose or reduced-fluence photodynamic therapy currently represents the most established interventional option [28].

Canrenone was used as adjunctive mineralocorticoid receptor antagonist during the acute episode. Its selection was based on pharmacokinetic considerations, including its active receptor antagonism and prolonged elimination half-life, permitting once-daily administration [29,30]. The favorable anatomical response observed in this case should primarily be interpreted in the context of corticosteroid withdrawal and control of the underlying inflammatory disease rather than attributed to mineralocorticoid receptor blockade alone. Although no recurrence was observed during the available follow-up, the relatively limited observation period does not exclude subsequent recurrence of either CSCR or inflammatory activity, supporting the need for continued clinical and multimodal imaging surveillance.

## 6. Conclusions

Steroid-induced CSCR is a clinically relevant and potentially underrecognized complication in patients with uveitis, particularly with PICCPs, undergoing corticosteroid therapy. Its recognition is critical, as subretinal fluid may mimic persistent inflammatory activity and lead to inappropriate management. Multimodal imaging plays a central role in distinguishing choriocapillaris-driven inflammation from choroidal hyperpermeability, enabling accurate diagnosis. Early identification should prompt timely therapeutic adjustment, including corticosteroid tapering and the introduction of steroid-sparing strategies. In selected cases, targeted modulation of the mineralocorticoid pathway may represent a useful adjunct to facilitate resolution of subretinal fluid.

## Figures and Tables

**Table 1:** Multimodal imaging features distinguishing primary inflammatory choriocapillaropathies (PICCPs) from steroid-induced central serous chorioretinopathy (CSCR). OCT: optical coherence tomography; FAF: fundus autofluorescence; FA: fluorescein angiography; ICGA: indocyanine green angiography.

Imaging Modality	PICCPs (Inflammatory Choriocapillaropathies)	Steroid-Induced CSCR
**OCT**	Outer retinal disruption (ellipsoid zone), ischemic damage; RPE alterations; possible bacillary layer detachment; band-like hyperreflective changes in superficial choroid; vitreous hyperreflective dots (inflammatory cells)	Dome-shaped serous retinal detachment; usually preserved outer retinal architecture (early stages); absence of inflammatory vitreous signs
**Choroid (EDI-OCT)**	Variable; may reflect inflammatory involvement	Increased choroidal thickness (pachychoroid phenotype)
**FAF**	Active lesions: hyperautofluorescent (lipofuscin accumulation, photoreceptor damage); Inactive lesions: hypoautofluorescent (RPE atrophy); possible “cockade” pattern in ampiginous choroiditis	Acute phase: hypoautofluorescent due to masking by subretinal fluid ± focal hyperautofluorescent spots (photoreceptor debris); Chronic phase: granular mixed pattern due to RPE dysfunction
**FA**	Early hypofluorescence (choriocapillaris non-perfusion); late staining or mild hyperfluorescence	Focal RPE leakage (“inkblot” or “smokestack” pattern)
**ICGA**	Areas of choriocapillaris hypoperfusion (persistent hypofluorescence)	Choroidal hyperpermeability (diffuse or focal hyperfluorescence)

## Data Availability

The original contributions presented in this study are included in the article. Further inquiries can be directed to the corresponding author.
